# Interpretive Frames to Understand the Contribution of Digital Exclusion to Health Inequalities: Hermeneutic Review

**DOI:** 10.2196/98204

**Published:** 2026-11-13

**Authors:** Diana Bright, Chiara Tuveri, Chrysanthi Papoutsi, Sara Shaw, Jackie van Dael, Nadia Swann, Scott Tandy, Catherine Naamani, Jocelle Lovell, Alisha Davies

**Affiliations:** 1 Research Data & Digital Directorate, Public Health Wales, No 2 Capital Quarter, Tyndall Street, Cardiff, Wales, CF10 4BZ, United Kingdom, 44 02920104699; 2 Nuffield Department of Primary Care Health Sciences, University of Oxford, Oxford, England, United Kingdom; 3 Newydd Housing Association (1974), Cardiff, Wales, United Kingdom; 4 Displaced People in Action, Cardiff, Wales, United Kingdom; 5 Cwmpas, Cardiff, Wales, United Kingdom

**Keywords:** digital exclusion, digital inclusion, hermeneutic review, health inequalities, social determinants of health

## Abstract

**Background:**

Digital exclusion is a multidimensional and relational process shaped by interactions among individuals, technologies, institutions, and wider social structures. Research on digital exclusion has expanded significantly in recent years, largely due to the COVID-19 pandemic and the rapid digitalization of health, welfare, and public services. Yet, despite this growth, conceptualizations of digital exclusion remain fragmented, limiting a comprehensive understanding of how digital exclusion contributes to the creation or intensification of health inequalities.

**Objective:**

This hermeneutic review aims to develop an interpretive understanding of how digital exclusion has been conceptualized across diverse bodies of literature in relation to health inequalities.

**Methods:**

We conducted a hermeneutic review using keyword searches and snowballing across PubMed, Scopus, and supplementary sources published between January 2020 and July 2025. Analysis involved repeated reading, note-taking, and comparative interpretation of each text’s disciplinary field, theoretical orientation, and underlying assumptions about how digital exclusion is framed in relation to health inequalities. Knowledge users contributed throughout to ensure the developing interpretations remained relevant to policy, practice, and lived experience.

**Results:**

Thirty-one publications were included, spanning multiple disciplines and theoretical orientations. Five interpretive frames were identified: (1) inequality, which conceptualizes digital exclusion as arising from unequal social, economic, and material conditions; (2) power and social injustice, which highlights how institutional rules, governance arrangements, cultural norms, and service pathways condition who is recognized or supported, showing how digital systems can reproduce existing social hierarchies; (3) life transitions, which illustrates how exclusion shifts during periods of disruption or change, emphasizing the fluctuating conditions and vulnerabilities associated with major life changes; (4) psychobehavioral, which focuses on psychological, cognitive, and emotional responses to digital demands; and (5) sociotechnical misalignment, which frames exclusion as arising when digital systems do not align with people’s routines or everyday contexts. These interpretive frames show how digital exclusion is understood through different yet overlapping explanations that emphasize structural conditions, institutional practices, life circumstances, individual experiences, and service and system design.

**Conclusions:**

This hermeneutic review identifies 5 interpretive frames that integrate diverse disciplinary perspectives on how digital exclusion contributes to health inequalities. The frames clarify the multiple, intersecting processes through which exclusion is produced and reproduced, helping to explain its persistence and uneven impacts. Our findings highlight the need for future research that examines structural and institutional mechanisms alongside lived experience and for digital health policy and practice to move beyond access- and skill-focused solutions toward approaches that address the multiple and interacting causes of digital exclusion.

## Introduction

The rapid digitalization of health, welfare, and public services has transformed how people access care, information, and support [1]. Digital technologies offer new opportunities to improve efficiency, access, and self-management. However, they also risk exacerbating existing health inequalities when designed, implemented, or delivered in ways that do not align with the resources, circumstances, or needs of different population groups [2]. Evidence from the pandemic period shows that rapid shifts to digital services were not evenly distributed. Without targeted mitigation, these changes can deepen disadvantages among communities facing structural barriers such as socioeconomic deprivation, limited connectivity, language barriers, and inadequate institutional support for digital engagement [3,4]. Concerns about digital exclusion have therefore become increasingly prominent in public health, social policy, and health services research, particularly in the context of accelerated digital adoption following the COVID-19 pandemic [5-7].

Over the past 2 decades, research on digital exclusion has undergone a substantial conceptual evolution. Early work centered on individual-level explanations, distinguishing between “users” and “nonusers” and attributing exclusion to personal access, motivation, or digital skills [8,9]. Research then moved beyond these narrow distinctions to highlight that inequalities unfold across multiple levels, encompassing access (first-level divide), skills and use (second-level divide), and the outcomes that individuals can obtain from digital engagement (third-level divide) [10-12]. This foundational work established that digital exclusion is closely intertwined with existing patterns of social and economic inequality, shaped by variations in resources, social position, and institutional contexts [13-15]. As digital technologies became more deeply integrated into everyday life, researchers increasingly recognized that exclusion arises from relational and structural conditions, not just from individual capabilities [16,17].

Building on this foundation, recent work conceptualizes digital exclusion as a dynamic and context-dependent phenomenon. Contemporary approaches emphasize that access, skills, use, and outcomes are interrelated, but they also highlight that digital participation is shaped by the design and governance of digital systems and by the social, organizational, and cultural environments in which they are used [17-19]. This work underscores that digital exclusion reflects the alignment (or misalignment) between digital systems and the diverse circumstances and needs of individuals or population groups [20,21]. For instance, a person may have access to online health services yet still experience exclusion if the platforms are inaccessible, difficult to navigate, or misaligned with their health literacy, language, or health needs [22]. These perspectives position digital exclusion as a relational phenomenon arising through interactions between people, technologies, and the wider social and institutional context. This also raises important questions about how responsibility for exclusion is conceptualized within the literature and where solutions are thought to reside, whether at the level of individuals, organizations, technologies, or broader social systems.

Previous reviews have mapped the evolution of digital exclusion research [23], developed a taxonomy of vulnerable groups [24], synthesized diverse theoretical traditions [25], and identified dimensions, barriers, and determinants of digital inequality [26-29]. This work demonstrates the breadth and complexity of the field and the diversity of perspectives used to frame digital exclusion. Despite these important contributions, less attention has been paid to how different theories, concepts, and assumptions are mobilized to explain the relationship between digital exclusion and health inequalities and how these diverse perspectives relate to one another [25,29,30]. As a result, literature provides diverse explanations of digital exclusion but offers limited insight into how these explanations connect, overlap, or diverge. These different ways of conceptualizing digital exclusion have important implications for how responsibility is assigned, how health inequalities are explained, and what forms of intervention are prioritized.

To address this gap, we conducted a hermeneutic analysis of the literature and developed 5 interpretive frames that reveal how different bodies of literature explain exclusion, assign responsibility, and suggest different intervention approaches. In doing so, the review provides a more coherent account of the field and its implications for research, policy, and practice.

## Methods

### Study Design

We conducted a hermeneutic review to develop an interpretive understanding of how digital exclusion has been framed in relation to health inequalities. The review involved a cyclical engagement with the literature, in which searching, reading, interpretation, and refinement of the focus proceeded iteratively as understanding deepened [31]. We approached relevance as something that became clearer through sustained engagement with the literature and ongoing comparison across texts.

The aim was not to provide an exhaustive account of all relevant studies but to bring together and interpret different ways the literature makes sense of digital exclusion within the context of health inequalities. Through this process, we developed interpretive frames that help organize and clarify the range of theoretical and analytical resources present in the literature. The protocol for the review was registered with the Open Science Framework [32]. Ethical approval was not required because this study comprised a hermeneutic review of published literature and involved neither human participants nor the collection of new data.

### Integrated Knowledge Translation

We used an integrated knowledge translation approach to support interpretation and ensure the review remained grounded in policy and practice realities [33]. This involved ongoing engagement with a knowledge users (KUs) advisory panel consisting of policymakers, practitioners, and community representatives, working with digitally excluded groups (eg, older adults, people with visual impairments, refugees, and asylum seekers). They contributed during the interpretive stages of the review through online meetings, discussions, and commentary, helping the research team reflect on emerging interpretations, challenge assumptions, and ensure that the developing frames remained relevant to policy, practice, and community experiences of digital exclusion.

### Searches

We began with an initial scoping phase to explore the breadth of the literature and gain a better understanding of the key concepts and terminology used in this field. This informed the subsequent searches and helped refine the developing focus. For example, prior work conducted by members of the research team and early scoping during protocol development highlighted the relevance of theories of capital, particularly Bourdieu’s [34] work. As searching and reading progressed, this process drew attention to the concept of digital capital and its recurring use in explanations of digital exclusion. Subsequent scoping searches included terminology around capital-based theories. Once the research team agreed on their relevance, these concepts were incorporated into the keyword strategy and informed the development of academic literature searches.

Electronic database searches were conducted in PubMed and Scopus for literature published between January 2020 and July 2025. This time frame was chosen to capture the wave of post–COVID-19 digital transformation across health and public services, which increased reliance on digital technologies and accelerated the adoption of digitally enabled models of care and service delivery. The intention was not to exclude earlier foundational work but to focus on contemporary conceptualizations of digital exclusion that have shaped the digital transformation of services. While insights from this period are not always directly transferable, they have significantly influenced contemporary policy discussions, service redesign, and digital front door approaches across the United Kingdom. The search strategy is available in Multimedia Appendix 1.

Additional searching involved backward and forward citation tracking, targeted searches of relevant technical agencies for gray literature, and sources recommended by KUs if aligned with the developing focus of the review. Although potentially relevant gray literature sources were examined, none met all inclusion criteria and were therefore not included in the review.

### Screening and Text Selection

Abstracts often provided limited insight into the conceptual or theoretical contributions of papers; therefore, broad criteria were applied during title and abstract screening, with inclusion decisions refined through in-depth reading. Screening was conducted in Rayyan (Qatar Computing Research Institute) by 2 reviewers (DB and CT). A calibration exercise on a 10% (76/760) sample helped establish a shared understanding of relevance, after which records were screened independently. Agreements, disagreements, and uncertainties were discussed within the research team to refine a shared interpretive understanding of relevance and to ensure consistency in the application of the inclusion and exclusion criteria (Textbox 1).

Following screening in Rayyan, full-text papers were retained when they contributed to an understanding of the mechanisms, processes, or contextual conditions through which digital exclusion operates in relation to health inequalities. Papers were considered conceptually relevant when they moved beyond describing the prevalence, consequences, or correlates of digital exclusion and provided theoretical insights into how exclusion is produced, experienced, or mitigated. Included papers also needed to engage with digital exclusion in ways that were relevant to health, health care, well-being, or health inequalities. Consistent with the hermeneutic approach, assessment of eligibility continued throughout the review. Searching, screening, reading, and eligibility assessment continued until additional sources no longer contributed substantively to new ways of understanding the topic. Final inclusion decisions were informed through iterative discussion and data charting within the research team. Inclusion and exclusion criteria are summarized in Textbox 1.

Textbox 1. **Inclusion and exclusion criteria for the screening process.**

**Inclusion criteria**

- Provided a conceptual or theoretical contribution through the use, development, application, or critique of frameworks, models, theories, or explanatory concepts on digital exclusion or inclusion
- Provided a conceptual or empirical contribution that helps explain how digital exclusion operates through relevant mechanisms, processes, experiences, relationships, or contextual conditions
- Included a health-related dimension, either through explicit links to health, health care, well-being, or health inequalities, or through consideration of social determinants of health (SDOH)
- Contributed to understanding digital exclusion among structurally disadvantaged or underrepresented populations, including those experiencing multiple and intersecting forms of disadvantage

**Exclusion criteria**

- Fell outside the scope of the review, including studies published before 2020, non-English records, inaccessible full-text, protocols, theses, or studies focused on general technology use without relevance to digital exclusion and health
- Provided only descriptive accounts of barriers, digital service use, health outcomes, or digital engagement without contributing conceptual, theoretical, or interpretive insights into how digital exclusion is produced, experienced, or reproduced
- Lacked a health-related dimension, including explicit or implicit links to health, health care, well-being, health inequalities, or relevant SDOH

### Data Analysis and Synthesis

Analysis involved iterative cycles of charting, close reading, note-taking, and comparisons across texts. For each text, we noted how it linked causes, mechanisms, and outcomes and how it framed digital exclusion in relation to health and inequality.

As part of this process, we examined each text in relation to three elements: (1) its disciplinary or field positioning, (2) the theoretical orientations it drew upon, and (3) its underlying assumptions about what influences exclusion and where responsibility for change is located (eg, within individuals, institutions, or wider structures). These aspects allowed us to distinguish between similarities and differences across the included papers as our understanding evolved.

Comparisons across texts focused on identifying recurring explanatory patterns as well as points of divergence. Papers were iteratively grouped and regrouped according to shared underlying assumptions, theoretical orientations, and explanations of how digital exclusion was produced and linked to health inequalities. Emerging interpretations were discussed within the research team and refined through continued engagement with the literature and input from KUs.

Through this process, we developed a set of interpretive frames that captured recurrent ways in which digital exclusion was conceptualized in relation to health inequalities. These 5 frames are best understood as overlapping interpretive perspectives rather than fixed categories, and papers often reflected elements of more than one frame. The resulting frames are presented in the *Results* section.

### Researcher Positionality

The review was undertaken by an interdisciplinary team with expertise spanning public health (DB, CT, and AD), digital health (DB, CT, CP, SS, JvD, NS, and AD), and health services research and social science (CP, SS, JvD, and NS), alongside public research partners (ST, CN, and JL), who contributed practice- and community-based perspectives informed by experience in housing, community development, and advocacy with underserved populations. Several members of the team had prior experience addressing and witnessing the consequences of digital exclusion in policy, practice, and community settings.

The review was conducted from a health inequalities perspective, reflecting a shared interest in understanding how digital exclusion contributes to health inequalities and a view that greater visibility of this relationship is necessary to inform action. We recognize that these experiences influenced the questions we asked of the literature and the interpretations we found most meaningful. As professionals working largely within digitally enabled environments, we are also mindful of our relative digital privilege compared with many of the populations discussed in the literature. Engagement with KUs brought additional insights from ongoing work with digitally excluded communities, helping to ensure that interpretations remained sensitive to experiences and forms of exclusion that may not be fully represented within the academic literature.

## Results

### Overview

Across the 31 academic publications included in the review, we observed wide variation in how digital exclusion was approached, explained, and connected to health inequalities. Figure 1 provides an overview of how publications entered the review at different stages, demonstrating the iterative and exploratory nature of our search process.

**Figure 1.**
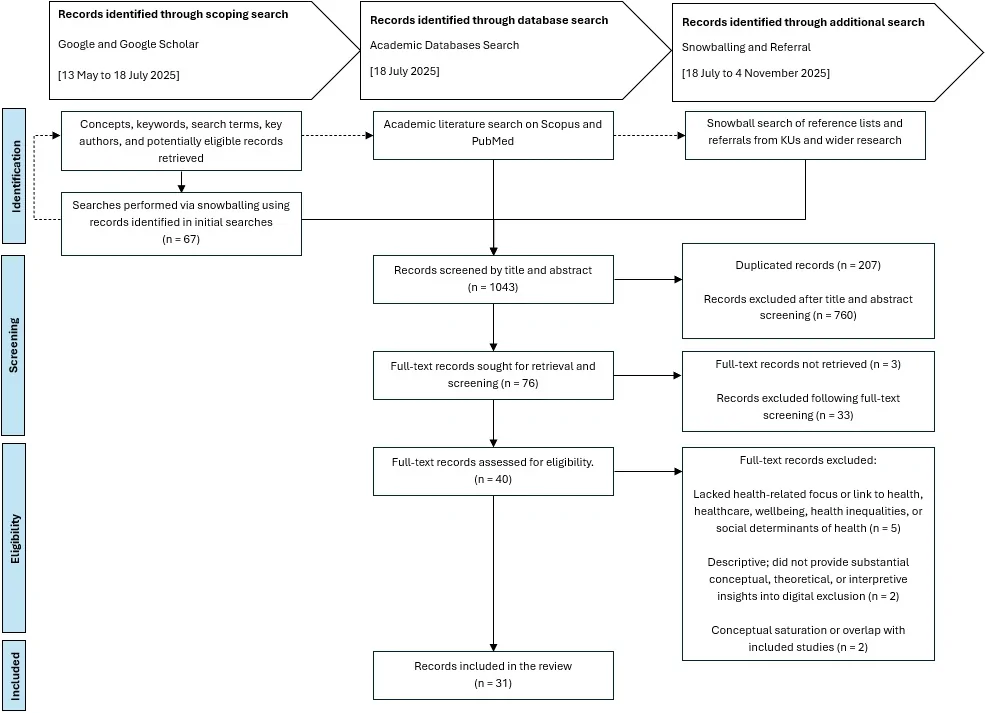
Study flowchart. KU: knowledge user.

Building on the interpretive analysis described above, we identified a set of interpretive frames that capture recurring ways in which digital exclusion is conceptualized in relation to health inequalities across different disciplinary fields and theoretical orientations. Across theoretical orientations, we identified 6 underlying assumptions that shaped how papers framed the nature of digital exclusion and where responsibility for addressing it was located. These assumptions were not always explicit but were inferred through repeated engagement with the literature and informed the development of the 5 interpretive frames presented below. They included *intersectionality perspectives*, emphasizing that overlapping characteristics such as age, gender, ethnicity, or disability can compound disadvantages; *multilevel explanations*, linking individual, community, organizational, and societal factors; s*tructural inequality perspectives*, framing exclusion as rooted in systemic conditions such as poverty, racism, or poor institutional support; *situation-dependent vulnerability*, emphasizing how life transitions contribute to experiences of exclusion; *individual-deficit explanations*, focused on skills, literacy, confidence, or motivation; and *technological advancement* as a contributor to digital exclusion when technologies are inaccessible, complex, or poorly designed. The frames provide a structured way of presenting the patterns that emerged while acknowledging the overlaps between them. Characteristics of the included studies appear in Multimedia Appendix 2, and a summary of the 5 interpretive frames, highlighting their explanatory logics and representative examples, is displayed in Table 1.

**Table 1.** Summary of interpretive frames, explanatory logics, and example studies.^a^

Interpretive frames	Inequality	Power and social injustice	Life transitions	Psychobehavioral	Sociotechnical misalignment
Digital exclusion is viewed as...	A socially embedded phenomenon arising from unequal distributions of social, economic, and material resources. Digital exclusion replicates and reinforces broader patterns of structural inequality rather than individual deficits.	A manifestation of deeper ethical, cultural, and institutional injustices. It mirrors unequal power relations that shape whose needs, voices, and participation are recognized, supported, and valued.	A dynamic, context-dependent process shaped by life transitions, aging, and social expectations. It is a matter of autonomy, agency, and the ability to navigate digital norms across different stages of life.	A phenomenon shaped by psychological, cognitive, and behavioral factors that influence motivation, confidence, emotional responses, and perceived capacity to engage with digital technologies.	Barriers arise when digital services and pathways are designed around assumptions that do not reflect people’s everyday contexts, needs, and available support.
Positionality on digital exclusion as a mechanism contributing to health inequalities	Digital exclusion contributes to health inequalities by reproducing and amplifying existing patterns of social and economic disadvantage.	Health inequalities are understood as consequences of unequal power relations and institutional practices that differentially recognize, accommodate, and support participation. Digital exclusion reflects and reproduces these inequitable conditions.	Key life transitions affect people’s agency, autonomy, and ability to navigate increasingly digital health services, contributing to health inequalities.	Health inequalities may emerge when people are unequally positioned to manage the psychological, cognitive, and emotional demands associated with digital engagement, shaping their ability to access and benefit from digital services.	The mismatch between assumptions embedded within digital health services and people’s realities creates barriers that can reinforce health inequalities by limiting participation and access to care.
Example of research questions reflecting the explanatory focus of each frame	How do structural inequalities shape people’s opportunities, capacities, and everyday experiences of getting online?	How do power relations and institutional practices shape patterns of digital exclusion and determine who is able to access and benefit from digital participation?	How do key life events and transitions in the life course influence digital exclusion?	How do people experience, interpret, and respond to digital demands, and how do these responses contribute to digital exclusion?	How do design assumptions in digital health misalign with people’s everyday realities, limiting their ability to navigate and benefit from digital services?
Examples of theoretical orientations	Resources and appropriation theory [35]; Bourdieu’s theory of capital [36]; Digital capital [37]	Intersectionality theory [30,38]; Bourdieu’s theory of capital [36,37]; Digital capital [39]; Gaventa’s Powercube [40]	Life course perspective [41]; Bourdieu’s practice theory [42]	Archer’s theory of reflexivity [43]; Bourdieu’s practice theory [42,44]	Unified theory of acceptance and use of technology (UTAUT) [45]; technology acceptance model (TAM) [46]
Common disciplinary fields represented	Sociology [35,39]; Public health and health services [47]	Sociology [48]; Public health and health services [49,50]	Sociology [41,42]	Public health and health services [43]; Psychology [51];	Public health and health services [49,50,52]; information and communication technology (ICT) [45]
Underlying assumptions commonly reflected	Structural inequality [35,39,53]; multilevel [54]	Intersectionality [30,38]; structural inequality [55]	Situation-dependent [56]; structural inequality [57]	Multilevel [43]; individual deficit [46]	Individual deficit [58]; technological advancement [45,46]

a Disciplinary fields, theoretical orientations, and underlying assumptions are not exclusive to 1 interpretive frame. This reflects how these elements are mobilized differently in the included literature to explain structural, institutional, relational, behavioral, and contextual processes that shape digital exclusion.

### Inequality

In this frame, digital exclusion is understood as a consequence of unequal social and economic conditions that shape people’s opportunities for digital participation and, in turn, their ability to access digital resources that can influence health and well-being. Institutional practices, cultural expectations, and uneven access to resources create patterned differences in access, engagement, and outcomes [37,38,47,53,56,57,59]. Within this perspective, digital exclusion is interpreted as a distributional consequence of wider social inequality rather than a distinct problem. The same conditions that shape access to income, education, housing, and social support also influence opportunities for digital participation. Health inequalities therefore emerge because social disadvantage affects who can access and benefit from increasingly digital forms of health care, information, and support [7,30,36,44,51,56,59]. The assumption underpinning this frame is that more equitable digital participation requires tackling broader social and economic inequalities, including poverty, educational disadvantage, discrimination, and unequal access to resources [35,39,54,56,60].

Within the inequality frame, theory is used to explain how digital exclusion replicates and reinforces wider social inequalities through cumulative and self-reinforcing processes. Drawing on sociology, Ragnedda et al [39] describe an “inequality loop” whereby existing social disadvantage constrains digital access, skills, and use, while digital exclusion further deepens social and economic marginalization. Digital exclusion is therefore interpreted not as an isolated disadvantage but as part of a broader cycle through which inequalities become reproduced over time. Positioned within political science, Lamberti et al [35] draw on resources and appropriation theory to conceptualize digital exclusion as a socially produced process through which inequalities in resources are translated into differences in motivation, access, digital skills, and internet use. Their cross-national analysis further suggests that the extent of these inequalities is shaped by the digital maturity of the national context, with more digitally developed countries partially mitigating disadvantages for less educated groups. These studies conceptualize digital exclusion as a process through which preexisting social inequalities accumulate and become translated into unequal opportunities for digital participation.

These interpretations are reinforced by broader socioecological accounts of digital health inequalities. Western et al [47] frame digital exclusion as a multidimensional and socially patterned phenomenon, arising from interactions between individual capabilities, community resources, service design, and wider structural conditions. From this perspective, inequalities in housing, education, income, and social support shape both opportunities for digital engagement and the extent to which individuals can benefit from digital health services. McCosker and Tucker [54] illustrate how wider inequalities are experienced in practice as low-income households navigate financial constraints, shared resources, and increasing digital demands. These studies conceptualize digital exclusion not simply as a lack of technology or skills but as a mechanism through which broader social inequalities are reproduced and experienced in everyday life.

Across this frame, digital participation is understood as mirroring wider social inequalities rather than operating as a neutral or leveling force. Health inequalities develop because the resources and opportunities needed to participate in and benefit from digital society are themselves unevenly distributed. Interventions focused solely on improving access or digital skills are unlikely to mitigate digital exclusion unless they are accompanied by efforts to address poverty, education, social support, and the broader social determinants of health (SDOH).

### Power and Social Injustice

Within this frame, digital exclusion represents a form of social injustice produced through power imbalances that systematically marginalize certain groups [30,48-50,52,55,57]. While the inequality frame focuses primarily on how social and economic disadvantage shapes opportunities for participation, the power and social injustice frame examines the unjust institutional, cultural, and political processes that determine whose needs are recognized, whose participation is valued, and whose voices influence decision-making [40,55,61]. Health inequalities arise through unequal access to resources and, crucially, from power relations that shape which needs are accommodated and how different groups benefit from digital services and opportunities [7,19,30,43,56,57,60].

For instance, Gaventa’s Powercube framework [40], which has been widely applied to examine power relations across social contexts, has been used to explore the gendered digital divide. The framework highlights how visible (policy), hidden (gatekeeping and agenda-setting), and invisible (norms and assumptions about competence and legitimacy) forms of power shape influence, recognition, representation, and participation, marginalizing the experiences of certain population groups. From this perspective, digital exclusion exposes unfair power relations that determine who is seen, heard, and accommodated in digital participation. Patterns of inclusion and exclusion privilege certain groups while rendering others invisible or dependent.

Husain et al [30] extend this interpretation by proposing fundamental cause theory, intersectionality theory, and digital capital theory as lenses through which digital health disparities can be understood. Although each focuses on different mechanisms, together they reframe digital exclusion from a set of individual barriers to a product of unequal social relations. For example, difficulties accessing digital health services are interpreted not merely as consequences of low digital literacy but as reflections of broader inequities in income, education, social support, disability, race, and other intersecting disadvantages that shape people’s capacity to engage with digital systems. Digital exclusion can consequently both emerge from and contribute to wider health inequalities.

Dakin et al [50] bring this interpretation into the everyday operation of health care services through their theory of “digital candidacy.” They demonstrate how access to digital primary care is shaped by institutional judgments about who is deemed an appropriate or legitimate digital user. Routine organizational practices, including triage and administrative decision-making, can produce exclusion by actively determining who is allowed to be recognized and assisted within digital pathways.

The concept of “digital othering” introduced by Berg [60] further illustrates the role of recognition and marginalization in producing digital exclusion. Focusing on refugees in Germany, Berg shows how restricted internet access, exclusionary norms, and administrative barriers intersect with race, gender, and religion to shape participation and service navigation. These processes create unfair opportunities for participation and reproduce broader inequalities by privileging the needs and experiences of those whose identities are established as legitimate.

The digital rainbow model [19] similarly places digital exclusion within broader social and institutional arrangements. By conceptualizing digital conditions as determinants of health inequities, it highlights how governance structures, service organization, and wider social inequalities collectively shape opportunities for participation and access to digital benefits. This shifts attention away from individual barriers toward the wider conditions that enable or constrain participation.

Overall, the power and social injustice frame positions digital exclusion as a political and ethical issue rooted in unequal power relations and institutional practices. Health inequalities arise when digital systems and services systematically privilege some groups while marginalizing others. Addressing digital exclusion requires a fundamental change to the power dynamics, governance structures, and institutional arrangements that determine how digital opportunities are designed and distributed. This frame places responsibility at the institutional level, arguing that shifts in power and governance are necessary to create more equitable opportunities for participation in digital society, including within health contexts.

### Life Transitions

This frame explains digital exclusion as a fluid and context-dependent phenomenon that changes over the life course. Rather than being a fixed characteristic of individuals or groups, it is shaped by changes in individuals’ experiences across the life course [41]. Life events such as entering education, changes in employment, retirement, illness, bereavement, or shifts in caregiving responsibilities reshape individuals’ capacity, motivation, and opportunity to engage with digital systems [42,51,56,57]. Transitions may create points of vulnerability when digital infrastructures fail to accommodate shifting circumstances. Across a person’s life, multiple or recurring moments of change may cause digital exclusion to surface or intensify. The studies contributing to this frame are predominantly rooted in sociological inquiry and examine how life-changing circumstances, social roles, and everyday practices shape opportunities for digital engagement [41,42,47,59].

A life course perspective positions transitions as critical junctures that reshape autonomy, practices, and digital engagement. Faure et al [41] conceptualize transitions as “biographical ruptures” that reconfigure an individual’s “room for maneuver” within social and institutional environments. Drawing on life course theory and social exclusion theory, they illustrate how these transitions intersect with structural inequalities (eg, limited education, age-related disadvantage, etc), heightening vulnerability during periods of change. Digital exclusion is interpreted as a dynamic condition emerging from changing circumstances that alter one’s ability to navigate digitally mediated environments.

Gallistl et al [56], through a praxeological approach (eg, practice-based), reflect on how older people engage with digital technology in their everyday lives, instead of treating “use” and “nonuse” as fixed categories. Periods of transition are not just triggers for withdrawal but shifts in the sociotechnical configurations (eg, the relationship between humans and technologies) and everyday practices that shape digital engagement. Rather than nonuse being a static or deficient state, it is an ongoing practice negotiated within changing social roles, identities, relationships, and material arrangements.

Ehrari et al [42] bring a sociocultural and Bourdieusian lens to health-related digital management. Digital health technologies are conceptualized as a field in which engagement depends on individuals’ available “capital,” including education, cultural competence, and prior digital experience, as well as their “habitus” or established dispositions and routines shaping how people navigate health systems. Digital health literacy is framed as a form of cultural capital that enables health management. Although the study does not explicitly consider life course transitions, it illustrates how periods of increased reliance on health care systems may expose vulnerabilities in digital engagement and worsen the risk of exclusion, precisely when digital participation becomes most critical.

Studies in this frame highlight how digital exclusion is shaped by changing life circumstances, not fixed individual or group characteristics. Digital autonomy depends as much on social conditions and institutional adaptation as it does on individual capability, emphasizing that people may move in and out of digital exclusion across the life course as their circumstances, needs, and relationships with digital technologies evolve. Health inequalities occur when changing circumstances reduce people’s ability to engage digitally at times when access to services, information, or support is most needed. From this perspective, reducing digital exclusion requires flexible forms of support and service design that can adapt to people’s changing needs and circumstances over time.

### Psychobehavioral

Across this frame, psychological, cognitive, and behavioral processes influence how individuals perceive and respond to the demands of digital participation. Factors such as motivation, confidence, cognitive capacity, and coping resources contribute to patterns of engagement, avoidance, or withdrawal [42-44,46,51,58]. Exclusion does not only surface when digital opportunities are unavailable. The same digital requirements may be experienced and managed differently across individuals and populations, creating varying capacities and willingness to engage. The papers contributing to this frame are mainly positioned within the public health and health services fields and draw on psychological [43,51] and sociological theories [42,44] to examine how individuals experience and manage digital participation.

A central theme across this frame is that disengagement from digital technologies is not necessarily a consequence of limited capability. Fang et al [51] illustrate this through lifespan and socioemotional selectivity theories, showing how older adults may disengage from digital technologies because of anxiety, perceived irrelevance, or emotional prioritization, despite having the necessary skills. Digital exclusion is presented as how individuals evaluate digital participation in relation to their changing goals, values, and emotional orientations across the life course.

This interpretation is extended by Rybczynska-Bunt et al [43], who focus on the reflexive work required to navigate increasingly digital health services. Using Archer theory of reflexivity, they show that exclusion may emerge when institutional demands exceed individuals’ capacity to reflect, plan, and act. Their concept of “fractured reflexivity,” describes how overwhelming cognitive and emotional demands may result in behaviors such as digital passivity, withdrawal, or maladaptive engagement, particularly in already vulnerable individuals.

Fisk et al [62] broaden the focus from individual characteristics to the wider conditions that shape people’s capacity to engage with digital technologies. Drawing on Sen’s human capabilities approach, they challenge deficit-based interpretations of digital exclusion by conceptualizing confidence, agency, and meaningful participation as products of the opportunities people have to develop and exercise their capabilities in the digital arena. From this perspective, behaviors commonly associated with exclusion, such as anxiety, avoidance, and low self-efficacy, are responses to relational and organizational conditions (eg, mentoring, role modeling, and supportive service environments) that enable or constrain digital engagement.

A complementary perspective is offered by Chen et al [58], who frame digital engagement as the outcome of interactions between environmental demands and psychological responses. Using the stimulus-organism-response framework, they show how health care demands and digital interface complexity shape older adults’ cognitive and emotional states, including confidence, anxiety, and perceived burden. These psychological responses, in turn, influence patterns of digital engagement, assistance-seeking, or disengagement. Here, digital exclusion emerges when the cognitive and emotional demands associated with digital participation exceed people’s capacity or willingness to engage.

The studies included in this frame suggest that participation depends not only on what people are able to do but also on how they experience digital technologies, the burdens they impose, and the extent to which they feel capable of managing them. Health inequalities surface when these psychological and behavioral demands are unevenly experienced, particularly due to illness, physical and cognitive decline, or other forms of vulnerability. Efforts to reduce digital exclusion should concentrate on strengthening confidence, agency, and coping capacity while reducing unnecessary complexity and burden within digital environments.

### Sociotechnical Misalignment

Within this frame, digital exclusion is understood to arise when digital technologies, services, or pathways fail to align with the social, material, and institutional realities of people’s everyday lives [45,46,52]. Instead of viewing exclusion as the outcome of individual reluctance, this perspective focuses on the relationship between users and technologies, highlighting how engagement becomes difficult when digital services are designed around assumptions that do not reflect users’ needs, expectations, social roles, or available support [45,46,58]. Across public health and health services literature, engagement is influenced by how people can access, adopt, and integrate technologies into daily life, with digital nonuse often reflecting misalignment when digital systems demand more than users can reasonably manage [49,50,52,58]. Health inequalities may emerge when digital service design and expectations are better suited to the circumstances of some groups than others.

Chadwick et al [45] and Ruiz Figueroa et al [46] illustrate how technology adoption depends on more than individual willingness to engage. Drawing on the unified theory of acceptance and use of technology and the technology acceptance model, respectively, both studies show that perceptions of usefulness, ease of use, social support, and technology-related anxiety are shaped by broader social and relational contexts. Digital exclusion occurs when the demands embedded within technologies exceed the resources, support, or circumstances available to intended users. Technology adoption is therefore interpreted as a context-dependent process rather than a matter of individual choice.

While we positioned these papers predominantly within the power and social injustice frame, Pope et al [49], Dakin et al [50], and Sin et al [52] extend this interpretation by shifting attention from users’ experiences of digital technologies to the assumptions embedded within digital services themselves. Their work shows how digital service design, triage practices, and institutional processes shape how technologies are experienced and used in practice, influencing who can engage, benefit, and access support. Digital exclusion is therefore interpreted as a consequence of a misalignment between the expectations built into digital services and the actual circumstances, needs, and capabilities of the people they are intended to serve. Importantly, barriers to digital adoption do not arise only at the point when individuals evaluate a digital service but are also often produced earlier through inequitable design and triage practices that shape people’s opportunities and decisions to engage.

Across these studies, digital exclusion is interpreted as arising when assumptions embedded within digital technologies and services do not align with the circumstances, resources, and support available to intended users. Engagement, legitimacy, and access are not treated as fixed individual attributes but as outcomes of the relationship between people, technologies, and the contexts in which they are used. From this perspective, nonuse reflects a failure of fit between service expectations and users’ realities, not individual reluctance or incapacity. Health inequalities transpire when these mismatches disproportionately affect those already experiencing disadvantage, increasing the difficulty of accessing services and benefits that are increasingly delivered through digital platforms. Digital exclusion is therefore understood as arising through interactions between technologies, service arrangements, and the contexts in which they are used. Reducing digital exclusion therefore requires changes to the design, organization, and implementation of digital services to better accommodate diverse needs and contexts.

## Discussion

### Principal Findings

This hermeneutic review identified 5 interpretive frames through which digital exclusion is conceptualized in relation to health inequalities. While previous scholarship has established that digital exclusion is multidimensional, relational, and socially embedded [19,25,26,30], our review demonstrates that these characteristics are understood through distinct yet intertwining explanatory logics. These frames do not offer competing explanations; they illustrate different mechanisms through which digital exclusion is produced and reproduced, including the accumulation of disadvantage over time (inequality); the role of power, recognition, and institutional arrangements in shaping opportunities for participation (power and social injustice); disruption during key life transitions (life transitions); psychological and behavioral responses to digital demands (psychobehavioral); and the mismatch between technologies and users’ everyday contexts (sociotechnical misalignment). Our contribution lies in showing how these overlapping perspectives make sense of digital exclusion in different ways, highlighting particular mechanisms, assigning responsibility for change to different actors, and implying different approaches to addressing exclusion and its consequences. This interpretation also provides one explanation for tensions identified in eHealth research, where responsibility for addressing digital exclusion is positioned differently across individuals, professionals, organizations, and wider systems. This helps explain why digital exclusion persists, how it contributes to health inequalities through multiple pathways, and why interventions that focus on a single dimension (eg, access alone) frequently produce limited impact [30,47].

Although our interpretive frames operate as complementary perspectives, they also reveal important differences in how the included literature conceptualizes digital exclusion and its link to health inequalities or SDOH. These differences become visible in both the focus of each frame and in how shared theories are used. For instance, Bourdieusian perspectives were applied across several studies that draw on concepts of capital, habitus, and practice. Within the psychobehavioral and life transitions frames, these concepts help explain how dispositions, routines, and embodied experience shape everyday digital engagement [42,44]. In contrast, within the inequality and power and social injustice frames, the same concepts are used to highlight patterned distributions of advantage and how exclusion is reproduced through institutional and structural conditions over time [35-37,39,59]. This demonstrates that the same theoretical orientation can help explain both what individuals experience in their everyday lives and the broader social conditions that shape those experiences.

These differences are also reflected in where the frames locate mechanisms through which exclusion is produced. The inequality and power and social injustice frames place greater emphasis on wider social and institutional conditions [36,48,52], whereas the life transitions frame focuses on how these conditions are experienced and negotiated through changing circumstances across the life course [41]. By contrast, the psychobehavioral and sociotechnical misalignment frames focus more on what happens when people try to use digital services in practice, such as whether they feel confident, anxious, overwhelmed, or supported, and whether digital technologies fit into their everyday routines [43,45]. These contrasting perspectives lead to different ways of understanding health inequalities as a consequence of digital exclusion. The more structural frames (inequality and power and social injustice) interpret inequalities as rooted in enduring social patterns and institutional arrangements, whereas the more individually oriented and relational frames (life transitions, psychobehavioral, and sociotechnical misalignment) emphasize the ways exclusion can emerge when people’s needs, capabilities, or circumstances conflict with the design and expectations of digital services. Viewed in relation to one another, these perspectives highlight that no single explanation is sufficient on its own. Digital exclusion tends to be most persistent where everyday difficulties experienced by individuals overlap with the wider-system level barriers and long-standing social inequalities [30,35,38,39,43,47,50,51,54]. This reinforces the need for approaches that consider both lived experience and the broader contexts through which exclusion is produced.

Across the 5 interpretive frames, the literature also shows that digital systems are now embedded within many of the wider conditions that shape people’s health, including access to services, work and welfare opportunities, social support, and the organization of public services [19,47,49,50]. As more aspects of daily life become mediated by digital processes, the consequences of exclusion extend beyond the inability to use specific tools or platforms. Being digitally excluded can affect whether people are able to navigate health care, manage long-term conditions, access entitlements, communicate with services, or avoid stress created when services are digital-by-default [19]. Our work strengthens the view that digital participation now plays a fundamental role in shaping health-relevant opportunities and conditions and that the consequences of digital exclusion are likely to intensify as digital participation becomes increasingly central to modern life [63].

### Implications for Practice and Future Directions

Our interpretive frames provide a way of understanding the different mechanisms through which digital exclusion is explained and addressed across the literature. By making these mechanisms explicit, they can help researchers, policymakers, and practitioners identify the assumptions underpinning existing interventions, recognize dimensions of exclusion that may be overlooked, and develop more comprehensive responses that address multiple interacting causes of exclusion.

Efforts to mitigate digital exclusion within health contexts need to reflect the different perspectives identified across the 5 interpretive frames. Addressing structural disadvantage requires interventions that tackle poverty, education disparities, and cultural marginalization while also strengthening trusted social resources and relationships that can support digital participation [35,39,40,56]. Reducing institutional gatekeeping and ensuring alternatives to digital-by-default pathways similarly call for reforms to governance, participatory policy design, and investment in public infrastructure [40,48,52]. Research on life course dynamics and sociotechnical misalignment suggests that digital services must be flexible and responsive to shifting circumstances, offering nondigital options and continuity of care [41-43,51]. This includes greater attention to how technologies become embedded within organizational routines, care practices, and everyday contexts, and how mismatches between service expectations and users’ realities can create new forms of exclusion (eg, increased reliance on support when services are insufficiently attuned to people’s circumstances and care journeys) [64,65]. In addition, interventions that support confidence, reduce cognitive load, and provide stable interpersonal support through family and community links, or consistent relationships with health professionals can help people manage the emotional and cognitive demands of digital systems [43,51,58]. Designing effective and equitable interventions therefore requires combining these approaches and ensuring that digital systems and policies are responsive to the diverse circumstances in which people live and seek care.

The interpretive frames also have implications for evaluation and future research. Our analysis indicates that changes in aspects of digital engagement, such as confidence or perceived ease of use (psychobehavioral), address only a fraction of the mechanisms through which digital exclusion is produced. These improvements may have limited impact if affordability constraints persist (inequality), if support does not adapt to people’s circumstances during major life disruptions (life transitions), or if institutional pathways continue to make access conditional (power and social injustice and sociotechnical misalignment). Evaluations informed by our interpretive frames can therefore distinguish between superficial improvements and deeper, sustained changes in people’s ability to access and benefit from digital participation, helping identify for whom interventions work, in what contexts, and with what implications for health equity.

Research on digital exclusion continues to draw on diverse and often disconnected explanatory perspectives. As a result, there remains limited integration of understanding across structural, institutional, behavioral, and sociotechnical explanations of digital exclusion and their implications for health inequalities [25,29,30]. Evidence on whether digital inclusion efforts reduce health inequalities is also limited; most evaluations focus on digital uptake [66-68], not on health-relevant consequences such as continuity of care, burden of navigation, or whether some groups benefit more than others. There is a particular need for studies that examine institutional and system-level mechanisms, such as eligibility, triage, and digital-by-default service design [49,50,52], and how these mechanisms shape access to services and opportunities to benefit from digital participation [64,65]. While older adults are well represented in the existing literature, further gaps remain in understanding the experiences of children and young people, people with disabilities, rural communities, and those facing intersecting inequalities. Future research would benefit from mixed methods and longitudinal designs that capture how digital engagement evolves over time, stronger engagement with lived experience to understand how people navigate digital systems in practice, and evaluations that explicitly consider health inequalities alongside measures of digital participation.

### Strengths and Limitations

Compared to previous reviews [23,24] and a theoretical synthesis of digital inequality [25], our hermeneutic approach enabled a deeper interpretive synthesis of diverse conceptual work, allowing us to examine how digital exclusion is framed in relation to health inequalities. While people experiencing digital exclusion were not directly involved in the interpretive process, engagement with KUs helped bridge academic and practice perspectives by bringing insights from their work with digitally excluded communities. This helped ensure the interpretation was grounded in lived experience and relevant to policy and practice. The review also benefits from explicitly situating digital exclusion within the context of health inequalities, drawing on SDOH to make connections that are often implicit or fragmented across different disciplinary fields and theoretical orientations. As with all interpretive syntheses, the findings reflect our analytical judgment and the dataset we have been able to compile through iterative searches. Given the rapid pace of change in the field, future research may refine and build on our work as new forms of digital practice, service delivery, and exclusion emerge. While interpretations were refined through discussion across a diverse research team and KUs, researchers approaching the literature from different disciplinary or theoretical traditions may have identified alternative explanatory patterns or constructed frames differently. This does not detract from the contribution but highlights the need for further theoretically informed research examining the link between digital exclusion and health inequalities.

### Conclusions

This review offers 5 interpretive frames that bring together diverse disciplinary fields, theoretical orientations, and underlying assumptions to explain how digital exclusion is conceptualized in relation to health inequalities. Our work highlights that digital exclusion is understood through distinct yet overlapping explanations that emphasize different mechanisms, assign responsibility to different actors, and suggest different responses. Collectively, these frames portray digital exclusion as a dynamic and relational process shaped by structural conditions, institutional practices, life circumstances, individual experiences, and system design. These perspectives help explain why digital exclusion persists, why its effects are unevenly distributed, and how it can contribute to widening health inequalities when digital systems become central to accessing health care and public services. As services move further toward digital modes of delivery, supporting meaningful and equitable digital participation requires coordinated attention to service design, organizational practices, and the broader social conditions in which people live. Future theoretically informed research is essential for assessing the conditions under which digital transformation can reduce, rather than exacerbate, health inequalities.

## Supplementary material

10.2196/98204 Multimedia Appendix 1 Search strategy for Scopus and PubMed.

10.2196/98204 Multimedia Appendix 2 Characteristics and conceptual profiles of included studies.

## Abbreviations

KU: knowledge user
SDOH: social determinants of health
