# Supplementary material for: Interpretive Frames to Understand the Contribution of Digital Exclusion to Health Inequalities: Hermeneutic Review
Source: Online J Public Health Inform. 2026 Nov 13;18:e98204. doi: 10.2196/98204 (PMC13766660; doi:10.2196/98204)
Supplement: Supplementary file 1 [file ojphi-v18-e98204-s001.docx]

**Multimedia Appendix 1**

Search Strategy

The searches were applied to Scopus and PubMed and extracted on the same day 07/18/2025.

| **Search Concept** | **Set** | **Search Terms** |
| --- | --- | --- |
| **Database: Scopus** | | |
| Digital exclusion and IT | S1 | TITLE-ABS(("digital exclusion" OR "digital inclusion" OR "digital divide*" OR "digital division*" OR "digital vulnerabilit*" OR "digital povert*" OR "digital disparit*" OR "digital gap*" OR "digital disengag*" OR “digital engag*” OR "digital inequalit*" OR "digital inequit*" OR "digital equit*" OR "digital illiteracy" OR "digital literacy" OR "computer illiteracy" OR "ICT illiteracy" OR "ICT literacy" OR "computer skills" OR "ICT skills" OR "information and communicat* technolog*" OR "internet non-use*" OR "internet use*")) |
| Theories and concepts | S2 | TITLE-ABS((theor* OR "theoretical model*" OR "theoretical approach*" OR typolog* OR taxonom* OR "analytical approach" OR “analytical theory” OR classification OR framework* W/3 (theor* OR concept* OR model* OR construct*))) |
| Specific theories and concepts | S3 | TITLE-ABS(("Actor-Network Theory" OR ANT OR (Bourdieu AND "social capital") OR "Capability Approach" OR ("Critical Race Theory" AND ("digital exclusion" OR "digital divide")) OR ("Deprivation Theory" AND ("digital exclusion" OR "digital divide")) OR "Diffusion of Innovations" OR "Digital Capital Theory" OR "Digital Divide Theory" OR "Digital Governmentality" OR ("Feminist Technoscience" AND ("digital exclusion" OR "digital divide")) OR "Information Poverty Theory" OR "Resources of Appropriation Theory" OR "Social Construction of Technology" OR SCOT OR "Sociotechnical transitions" OR "Technology Acceptance Model" OR TAM OR "Unified Theory of Acceptance and Use of Technology" OR UTAUT)) |
| Health and wellbeing | S4 | TITLE-ABS((“health inequalit*” OR wellbeing OR "well-being" OR "mental health" OR "public health" OR "social care")) |
|  | S5 | S2 OR S3 |
|  | S6 | S1 AND S5 |
|  | S7 | S4 AND S6 |
|  | S8 | AND PUBYEAR > 2019 AND PUBYEAR < 2026 AND (LIMIT-TO (LANGUAGE,"English")) AND ( LIMIT-TO (SUBJAREA,"MEDI") OR LIMIT-TO (SUBJAREA,"SOCI") OR LIMIT-TO (SUBJAREA,"COMP") OR LIMIT-TO ( SUBJAREA,"PSYC" )) |
| **Database: PubMed** | | |
| Digital exclusion and IT | S1 | ("Digital Divide"[MeSH Terms] OR "Internet Use"[MeSH Terms] OR "Internet Access"[MeSH Terms] OR "Information Technology"[MeSH Terms] OR "digital exclusion"[Title/Abstract] OR "digital inclusion"[Title/Abstract] OR "digital divide*"[Title/Abstract] OR "digital division*"[Title/Abstract] OR "digital vulnerabilit*"[Title/Abstract] OR "digital povert*"[Title/Abstract] OR "digital disparit*"[Title/Abstract] OR "digital gap*"[Title/Abstract] OR "digital disengag*"[Title/Abstract] OR "digital engag*"[Title/Abstract] OR "digital inequalit*"[Title/Abstract] OR "digital inequit*"[Title/Abstract] OR "digital equit*"[Title/Abstract] OR "digital illiteracy"[Title/Abstract] OR "digital literacy"[Title/Abstract] OR "computer illiteracy"[Title/Abstract] OR "ICT literacy"[Title/Abstract] OR "computer skills"[Title/Abstract] OR "ICT skills"[Title/Abstract] OR "information and communicat* technolog*"[Title/Abstract] OR "internet non use*"[Title/Abstract] OR "internet use*"[Title/Abstract]) |
| Theories and concepts | S2 | ("Classification"[MeSH Terms] OR "theor*"[Title/Abstract] OR "theoretical model*"[Title/Abstract] OR "theoretical approach*"[Title/Abstract] OR "typolog*"[Title/Abstract] OR "taxonom*"[Title/Abstract] OR "analytical approach"[Title/Abstract] OR "analytical theory"[Title/Abstract] OR "Classification"[Title/Abstract] OR "theor* framework*"[Title/Abstract] OR "concept* framework*"[Title/Abstract] OR "construct* framework*"[Title/Abstract] OR "model* framework*"[Title/Abstract]) |
| Specific theories and concepts | S3 | ("Intersectional Framework"[MeSH Terms] OR "Social Capital"[MeSH Terms] OR "Diffusion of Innovation"[MeSH Terms] OR "Actor-Network Theory"[Title/Abstract] OR "ANT"[Title/Abstract] OR "Bourdieu social capital"[Title/Abstract:~3] OR "Bourdieus social capital"[Title/Abstract:~3] OR "Capability Approach"[Title/Abstract] OR "Critical Race Theory digital exclusion"[Title/Abstract:~3] OR "Critical Race Theory digital divide"[Title/Abstract:~3] OR "Deprivation Theory digital exclusion"[Title/Abstract:~3] OR "Deprivation Theory digital divide"[Title/Abstract:~3] OR "Diffusion of Innovations"[Title/Abstract] OR "Feminist Technoscience digital exclusion"[Title/Abstract:~3] OR "Feminist Technoscience digital divide"[Title/Abstract:~3] OR "Social Construction of Technology"[Title/Abstract] OR "SCOT"[Title/Abstract] OR "Sociotechnical transitions"[Title/Abstract] OR "Technology Acceptance Model"[Title/Abstract] OR "TAM"[Title/Abstract] OR "Unified Theory of Acceptance and Use of Technology"[Title/Abstract] OR "UTAUT"[Title/Abstract]) |
| Health and wellbeing | S4 | ("Health Inequities"[MeSH Terms] OR "Mental Health"[MeSH Terms] OR "Public Health"[MeSH Terms] OR "Social Support"[MeSH Terms] OR "health inequalit*"[Title/Abstract] OR "wellbeing"[Title/Abstract] OR "well-being"[Title/Abstract] OR "Mental Health"[Title/Abstract] OR "Public Health"[Title/Abstract] OR "social care"[Title/Abstract]) |
|  | S5 | S2 OR S3 |
|  | S6 | S1 AND S5 |
|  | S7 | S4 AND S6 |
|  | S8 | AND ((2020/1/1:2025/7/18[pdat]) AND (english[Filter])) |
