# Supplementary material for: Interpretive Frames to Understand the Contribution of Digital Exclusion to Health Inequalities: Hermeneutic Review
Source: Online J Public Health Inform. 2026 Nov 13;18:e98204. doi: 10.2196/98204 (PMC13766660; doi:10.2196/98204)
Supplement: Supplementary file 2 [file ojphi-v18-e98204-s002.docx]

**Multimedia Appendix 2**

Characteristics and conceptual profiles of included studies

| **Author, year, and country** | **Study design** | **Disciplinary fields ^a^** | **Theoretical orientations ^b^** | **Underlying assumptions ^c^** | **Interpretative Frame(s) ^d^** | **Conceptual framing of digital exclusion ^e^** | **Digital exclusion and its role in health inequalities ^f^** |
| --- | --- | --- | --- | --- | --- | --- | --- |
| Asmar et al 2020 [1], Belgium | Qualitative | Sociology | • Life course perspective  • Digital inequality and digital social support concepts  • Homophily in social networks | Structural inequality / Multilevel | Life transitions / Inequality | People’s digital engagement shifts across life stages and is shaped by structural inequalities; strong social support is essential for avoiding exclusion. | Acknowledges that social support mediates access to digital services including health services. |
| Asmar et al 2022 [2], Belgium | Literature review | Sociology | • Bourdieu’s theory of capital  • Corresponding fields model  • Miliband's classification on social exclusion | Structural inequality / Situation-dependent | Inequality / Power and Social Injustice | Digital exclusion is not a single condition but a set of diverse profiles, shaped by different combinations of disadvantage across access, skills, use, and outcomes, with no single dominant pathway. | Digital exclusion is framed as an interaction between social and digital factors that shapes well‑being, ranging from deep exclusion to deep inclusion. |
| Berg 2025 [3], Germany | Qualitative | Sociology | • Western ethnocentrism and coloniality reflections  • Social identity theory  • “The other” and “othering” concepts | Intersectionality / Multilevel | Power and Social Injustice / Inequality | Digital exclusion is rooted in power imbalances and institutional systems; migrants, refugees, and asylum seekers navigate unequal digital infrastructures that go beyond simple socioeconomic vulnerability. | Digital exclusion undermines refugees’ mental well‑being by reinforcing isolation and limiting access to support and connection. |
| Chadwick et al 2024 [4], UK | Qualitative | Information and Communication Technology (ICT) | • Unified Theory of Acceptance and Use of Technology (UTAUT)  • Intersectionality and digital intersectionality | Individual deficit / Technological advancement | Sociotechnical Misalignment / Inequality | Digital exclusion is explained as a failure of adoption influenced by perceived usefulness, effort, social norms, and facilitating conditions, with social deprivation and older age shaping these factors. | Highlights that older individuals and those in socially deprived areas lack access to digital health technologies and the skills to use them, which exacerbates health inequities. |
| Chan & Guan 2024 [5], Singapore | Quantitative | Sociology | • Bourdieu’s practice theory | Structural Inequality / Individual deficit | Inequality/ Psychobehavioral | Digital exclusion arises from unequal configurations of social, cultural, economic, and bodily capital that shape dispositions such as motivation and self‑efficacy, particularly influencing older adults’ attitudes and patterns of ICT use. | Among older and socially deprived populations, digital exclusion restricts well-being, independence, and access to digital health services, deepening health inequalities. |
| Chen et al 2025 [6], China | Qualitative | Public health and health services | • Stimuli-Organism-Response (SOR) framework | Individual deficit / Technological advancement | Psychobehavioral / Sociotechnical Misalignment | Older adults’ engagement with digital health tools is shaped by psychological and behavioral factors, particularly perceived usefulness and effort. | Digital exclusion among older and socially deprived populations worsens health inequities, particularly during COVID‑19, driven by limited access, low digital skills, negative attitudes, and low motivation. |
| Dakin et al 2024 [7], UK | Qualitative | Public health and health services | • Candidacy theory  • Digital candidacy  • User configuration theories  • Articulation theories  • Structuration theories  • Technology-enhanced strong structuration theory | Structural Inequality / Technological advancement | Power and Social Injustice / Sociotechnical Misalignment | The paper conceptualizes digital exclusion as institutionally mediated digital candidacy, whereby digital‑first healthcare favors ideal users and can deepen inequalities without multiple access pathways. | Digital triage can unintentionally deepen healthcare barriers, limiting access for digitally excluded groups and creating new risks within digital‑first health systems. |
| Davies et al 2021 [8], UK | Viewpoint | Public Health and Health Services | • Digital inverse care law | Structural Inequality / Multilevel | Power and Social Injustice / Inequality | Digital exclusion is framed through the digital inverse care law, whereby digital health systems systematically advantage those with least need and disadvantage those with greatest need unless wider social conditions are addressed. | Digital exclusion maps onto existing social gradients in health. |
| Ehrari et al 2022 [9], Denmark | Qualitative | Sociology | • Bourdieu’s practice theory | Structural inequality / Individual deficit | Psychobehavioral / Life transitions | Digital exclusion reflects socio‑cultural literacy barriers that become most acute during periods of illness and increased digital care use. | Limited digital health literacy, shaped by social factors such as capital and habitus, leads to poorer health management, higher costs, and unequal health outcomes. |
| Fang et al 2025 [10], China | Literature review | Psychology | • Motivational theory of lifespan development  • Socioemotional selectivity theory | Individual deficit / Situation-dependent / Multilevel | Psychobehavioral / Life Transitions | Digital exclusion in later life arises when demands become overwhelming during key transitions, with psychological and structural barriers compounding isolation and reduced support. | Digital exclusion worsens older adults’ health and well‑being by increasing isolation and limiting access to health information, care, and informed decision‑making. |
| Faure et al 2020 [11], Belgium | Qualitative | Sociology | • Life course perspective  • Digital exclusion and autonomy theories | Structural Inequality / Situation-dependent | Life Transitions | Digital exclusion is not a stable status but a process that unfolds as people move through different life stages, roles, and circumstances. | Shows how social determinants (education, employment, social support, life events) shape situational risks of digital exclusion. |
| Fersch et al 2025 [12], Denmark | Qualitative | Sociology | • Bourdieu’s theory of capital  • Digital capital | Structural Inequality | Inequality / Power and Social Injustice | Digital‑by‑default systems assume people can rely on social networks to manage digital access, while failing to account for rural contexts, which allows governance practices to reinforce spatial inequalities. | Lack of digital access increases health inequalities, but digital skills and social support can help protect against these effects. |
| Fisk et al 2022 [13], USA | Mixed-Methods | Public health and health services | •Human capabilities approach  • Strengths-based perspective  • Vulnerability, digital divide, and service inclusion concepts | Multilevel | Psychobehavioral / Power and Social Injustice | Digital inclusion is framed in terms of people’s ability to achieve valued outcomes (participation, autonomy, wellbeing, and health) rather than access alone; social and economic inequalities limit the conversion of digital access into benefits. | Digital exclusion undermines access to essential services and well-being, while inclusion supports resilience and autonomy. |
| Gallistl et al 2020 [14], Austria | Mixed-Methods | Sociology | • Critical and cultural gerontological perspective | Structural Inequality | Power and Social Injustice | Digital exclusion is produced through institutional and policy framings that construct older people as “non‑users,” rather than through individual deficits or choices. | Poor health among older adults, including sensory and cognitive limitations, is associated with lower internet use and increased digital exclusion. |
| Gallistl et al 2021 [15], Austria | Qualitative | Sociology | • Practice-Based perspective on gerontology  • Actor-network theory | Situation-dependent / Structural Inequality | Life transitions / Inequality / Power and Social Injustice | Explains digital exclusion as something enacted through older adults situated everyday practices of non‑use, with inequality shaping vulnerability. | Digital exclusion contributes to isolation and poorer well-being, with socio‑economic barriers and technophobia restricting access to health information and services. |
| Holmes & Burgess 2022 [16], UK | Qualitative | Sociology | • Relative deprivation theory  • Corresponding fields model  • Yate’s Digital-By-Default | Structural Inequality / Intersectionality | Inequality | Digital exclusion reflects poverty and structural disadvantage; skills or access fixes alone cannot help without tackling the wider socio-economic conditions that shape people’s digital engagement. | Digital exclusion is intertwined with poverty, housing, and education, as stress and insecurity reduce digital engagement and access to well-being‑supporting resources. |
| Husain et al 2022 [17], UK | Narrative review | Public health and health services | • Fundamental cause theory  • Digital capital theory  • Intersectionality theory | Structural Inequality / Intersectionality | Power and Social Injustice / Inequality | Digital exclusion is explained as the product of intersecting social disadvantages that compound to shape unequal access to and benefit from digital health technologies, rather than isolated individual factors. | Digital exclusion has contributed to widening health inequalities during digital health expansion. |
| Jahnel et al 2022 [18], Germany | Essay | Public health and health services | • Dahlgren-Whitehead rainbow model | Multilevel | Power and Social Injustice | Conceptualizes digital exclusion as a structural and intersectional process rooted in power, recognition, and institutional responsibility. | Digital determinants interact with SDOH to widen health disparities, as shown by COVID‑19 digital public health tools that disadvantaged people with limited access or skills. |
| Kuiler 2024 [19], USA | Conceptual paper | Political science | • Spinoza’s ethical egoism and conatus  • Grotius’ natural law and justice in the world policy  • Aristotle’s aretaic ethics | Structural Inequality | Power and Social Injustice | Growing digital, knowledge, and geographic divides deepen systemic exclusion as rights become commodified, framing digital inequality as an ethical governance issue rooted in power and justice. | Digital and knowledge divides shape who can access, benefit from, and be protected by digitally mediated health systems, reinforcing structural inequities. |
| Lamberti et al 2021 [20], Spain | Quantitative | Political science | • Resources and appropriation theory  • Bourdieu’s theory of consumption  • Tilly’s Theory of Durable Inequality | Multilevel / Structural Inequality | Inequality | Digital exclusion as a socially structured, education‑driven process shaped by unequal access, skills, and motivation, and moderated by a country’s level of digital development. | People with fewer educational opportunities are more likely to be digitally excluded, this can limit their access to useful information and services, including health‑related ones. |
| McCosker & Tucker 2025 [21], Australia | Qualitative | Sociology | • Collective capabilities  • Digital bootstrapping | Structural Inequality / Situation-dependent | Inequality | Digital exclusion is described as a socially and materially produced outcome of poverty, becoming more acute when household circumstances change. | Digital exclusion in low‑income households deepens health inequalities as poverty‑related constraints limit access to the digital services and capabilities needed to support health and well-being. |
| Pope et al 2023 [22], UK | Qualitative | Public health and health services | • Scott’s sociology of nothing  • Garfinkel’s ideas on marginalized categories  • Brekhus’ sociology of the unmarked | Structural Inequality /  Technological advancement | Power and Social Injustice / Sociotechnical Misalignment | Digital‑first policies overlook socio-technical realities; health technologies succeed only when aligned with user contexts, while institutional design and governance can obscure services and constrain meaningful acceptance. | Low visibility and inconsistent integration of NHS 111 online may lead to unequal navigation of urgent care, indirectly contributing to health inequalities. |
| Ragnedda & Ruiu 2020 [23], UK | Book | Sociology | • Bourdieu’s theory of capital  • Digital capital | Structural Inequality | Inequality / Power and Social Injustice | Unequal access to and accumulation of digital capital limits skills and benefits for disadvantaged groups, reproducing wider social inequalities and making digital exclusion a structural downstream effect. | Digital capital shapes people’s ability to access opportunities and resources, this has indirect implications for health inequalities and SDOH. |
| Ragnedda et al 2022 [24], UK | Quantitative | Sociology | • Bourdieu’s theory of capital | Structural Inequality | Inequality / Power and Social Injustice | Digital exclusion is described as a cumulative process where social disadvantage limits people’s digital access, skills, and use, and this exclusion then reinforces and deepens their social and economic disadvantage over time. | Digital inclusion is treated as a well-being determinant, requiring policy attention for vulnerable groups. |
| Ruiz-Figeroa et al 2024 [25], Spain | Systematic review | Social work | • Technology Acceptance Model (TAM) | Individual deficit / Technological advancement | Sociotechnical Misalignment / Psychobehavioral / Inequality | Digital exclusion is seen as the result of individual perceptions, skills, and anxieties, shaped by situational and social factors, that hinder older adults’ willingness or ability to adopt and use technology. | Poor health, low skills, and technology anxiety contribute to digital exclusion, reducing autonomy and access to care, especially during COVID‑19. |
| Rybczynska-Bunt et al 2024 [26], UK | Qualitative | Public health and health services | • Archer’s theory of reflexivity | Structural inequality / Multilevel / Situation-dependent | Psychobehavioral / Power and Social Injustice | Exclusionary design shapes who can participate, triggering psychological barriers that disproportionately burden disadvantaged groups, as uneven reflexivity and cognitive processes reinforce structural marginalization. | The introduction of remote and digital modalities in healthcare has widened health inequities, particularly affecting marginalized populations. |
| Salemink et al 2025 [27], The Netherlands | Literature review | Sociology | • Mutual shaping  • Remapping | Structural Inequality / Situation-dependent | Power and Social Injustice | Rural digitalization is shaped by longstanding spatial inequalities, as market‑driven strategies neglect remote areas; a justice lens reframes exclusion as issues of fairness, recognition and responsibility. | Rural digitalization is constrained by structural spatial inequalities, with investment favouring urban areas, leaving rural communities with poorer and more costly connectivity that deepens existing disadvantages. |
| Sharma & Ojha 2025 [28], India | Systematic review | Sociology | • Gaventa’s Powercube Framework | Structural Inequality / Multilevel | Power and Social Injustice | The gender digital divide reflects visible, hidden and invisible power structures, unequal infrastructure, exclusion from decision‑making, and patriarchal norms; women’s digital participation is constrained by systemic inequalities. | Gendered power imbalances drive digital exclusion, which reproduces broader social determinants of health such as unequal access to resources, participation, and opportunities. |
| Sin et al 2021 [29], Canada | Peer-reviewed conference paper | Public health and health services | • Digital Design Marginalization (DDM) | Structural Inequality / Technological Advancement | Power and Social Injustice / Sociotechnical Misalignment | Design choices fail to accommodate diverse needs, structurally pushing users away and undermining the conditions required for meaningful acceptance, regardless of skills or access. | Digital exclusion and marginalizing design undermine older adults’ well-being and reinforce inequalities by limiting access, participation, and social inclusion. |
| Tsatsou 2022 [30], UK | Qualitative | Sociology | • Intersectionality • Digital intersectionality | Intersectionality / Situation-dependent | Power and Social Injustice / Inequality | Digital disadvantage results from intersecting inequalities and social stigma that influence who has access, skills, and meaningful digital outcomes. | Digital tools can enhance independence, cognitive stimulation, and social participation for older adults and support ethnic minorities in navigating health systems. |
| Western et al 2025 [31], The Netherlands | Narrative review | Public health and health services | • Digital health divide theory • Digital rainbow model | Intersectionality / Multilevel | Inequality | Digital exclusion is explained as a patterned outcome of unequal social and economic conditions (e.g. socioeconomic status, age, geography, and disability) that shape access to, use of, and benefit from digital health technologies. | Unequal access, skills, and capacity to benefit from digital health tools reproduce, and can worsen, existing social and health inequities. |
| ^a^ Disciplinary field inferred from the paper’s topic, academic field, journal, and authors’ affiliations indicating the lens through which digital exclusion is approached.  ^b^ Broad conceptual orientations inferred from the theories and frameworks used to frame digital exclusion.  ^c^ Recurring patterns across the literature that informed interpretations of digital exclusion and its underlying causes.  ^d^ Interpretative classification of how digital exclusion or inclusion are framed in each paper. When multiple frames are listed, the first represents the primary interpretative frame applied in the paper; subsequent frames indicate secondary orientations.  ^e^ Conceptual framing was derived through reviewers’ interpretive synthesis of how digital exclusion was described and discussed within each paper.  ^f^ Explicit/implicit link to health inequalities or social determinant of health (SDOH). | | | | | | | |

References

1. Asmar A, Van Audenhove L, Mariën I. Social Support for Digital Inclusion: Towards a Typology of Social Support Patterns. Soc Incl. 2020;8:138–50. doi: [10.17645/si.v8i2.2627](https://doi.org/10.17645/si.v8i2.2627)
2. Asmar A, Mariën I, Van Audenhove L. No one-size-fits-all! Eight profiles of digital inequalities for customized inclusion strategies. New Media Soc. 2022;24:279–310. doi: [10.1177/14614448211063182](https://doi.org/10.1177/14614448211063182)
3. Berg M. Refugee Integration in Germany: The Interplay of Othering, Digital Exclusion, and Identity Negotiation. J Int Migr Integr. 2025;26 .doi: [10.1007/s12134-025-01238-0](https://doi.org/10.1007/s12134-025-01238-0)
4. Chadwick H, Laverty L, Finnigan R, Elias R, Farrington K, Caskey F, et al. Engagement With Digital Health Technologies Among Older People Living in Socially Deprived Areas: Qualitative Study of Influencing Factors. JMIR Form Res. 2024;8:e60483–e60483. doi: [10.2196/60483](https://doi.org/10.2196/60483)
5. Chan C, Guan C. Digital Inclusion of Senior Citizens: A Practice Theory Approach. 2024. Hawaii International Conference on System Sciences 2024 (HICSS-57); 6.
6. Chen Y, Yin Q, Zhou T, Gu S, Xie L, Xiao M, et al. Digital Inclusion Pathways and Influencing Factors Among Older Adults in Outpatient Settings: A Grounded Theory Study. Clin Interv Aging. 2025;20:701–16. doi: [10.2147/CIA.S518045](https://doi.org/10.2147/CIA.S518045)
7. Dakin FH, Rybczynska-Bunt S, Rosen R, Clarke A, Greenhalgh T. Access and triage in contemporary general practice: A novel theory of digital candidacy. Soc Sci Med. 2024;349:116885. [doi: 10.1016/j.socscimed.2024.116885](https://doi.org/10.1016/j.socscimed.2024.116885)
8. Davies, A. R., Honeyman, M., & Gann B. Addressing the digital inverse care law in the time of COVID-19: potential for digital technology to exacerbate or mitigate health inequalities. J Med Internet Res. 2021;23:e21726. [doi:10.2196/21726](https://doi.org/10.2196/21726)
9. Ehrari H, Tordrup L, Müller SD. The Digital Divide in Healthcare: A Socio-Cultural Perspective of Digital Health Literacy. In Bui TX, editor, Proceedings of the 55th Annual Hawaii International Conference on System Sciences, HICSS 2022. University of Hawai'i at Manoa. 2022. p. 4097-4106.
10. Fang Y, Shao Y, Wang M. The Involuntary Experience of Digital Exclusion Among Older Adults: A Taxonomy and Theoretical Framework. Am Psychol. 2025; 30. doi: [10.1037/amp0001502](https://psycnet.apa.org/doi/10.1037/amp0001502)
11. Faure L, Vendramin P, Schurmans D. A situated approach to digital exclusion based on life courses. Internet Policy Rev. Alexander von Humboldt Institute for Internet and Society; 2020;9:1–18. doi: [10.14763/2020.2.1475](https://doi.org/10.14763/2020.2.1475)
12. Fersch B, Noe E, Thuesen A, Langer B. The Rural Risk of Digital Exclusion: A case study of municipal digital health and social care services in Denmark. SSM - Qual Res Heal. 2025;7:100537. doi: [10.1016/J.SSMQR.2025.100537](https://doi.org/10.1016/J.SSMQR.2025.100537)
13. Fisk R, Gallan A, Joubert A, Beekhuyzen J, Cheung L, Russell-Bennett R. Healing the Digital Divide With Digital Inclusion: Enabling Human Capabilities. J Serv Res. 2022;26:109467052211401. doi: [10.1177/10946705221140](https://doi.org/10.1177/10946705221140148)
14. Gallistl V, Rohner R, Seifert A, Wanka A. Configuring the Older Non-User: Between Research, Policy and Practice of Digital Exclusion. Soc Incl. 2020;8:233. doi: [10.17645/si.v8i2.2607](https://doi.org/10.17645/si.v8i2.2607)
15. Gallistl V, Rohner R, Hengl L, Kolland F. Doing digital exclusion – technology practices of older internet non-users. J Aging Stud. 2021;59:100973–100973.doi: [10.1016/j.jaging.2021.100973](https://doi.org/10.1016/j.jaging.2021.100973)
16. Holmes H, Burgess G. Digital exclusion and poverty in the UK: How structural inequality shapes experiences of getting online. Digit Geogr Soc. 2022;3:100041. doi: [10.1016/j.diggeo.2022.100041](https://doi.org/10.1016/j.diggeo.2022.100041)
17. Husain L, Greenhalgh T, Hughes G, Finlay T, Wherton J. Desperately Seeking Intersectionality in Digital Health Disparity Research: Narrative Review to Inform a Richer Theorization of Multiple Disadvantage. J Med Internet Res. 2022;24:e42358. [doi: 10.2196/42358](https://doi.org/10.2196/42358)
18. Jahnel T, Dassow H-H, Gerhardus A, Schüz B. The digital rainbow: Digital determinants of health inequities. Digit Heal. 2022;8:205520762211290. doi: [10.1177/205520762211290](https://doi.org/10.1177/20552076221129093)
19. Kuiler EW. Bridging digital and knowledge divides: Concepts, frameworks and ethical concerns. Policy and Internet. 2024;16:222–41. doi: [10.1002/poi3.400](https://doi.org/10.1002/poi3.400)
20. Lamberti G, López-Sintas J, Sukphan J. The social process of internet appropriation: Living in a digitally advanced country benefits less well-educated Europeans. Telecomm Policy. 2021;45:102055. doi: [10.1016/j.telpol.2020.102055](https://doi.org/10.1016/j.telpol.2020.102055)
21. Mccosker A, Tucker J. Bootstrapping the digital divide: a situational analysis of digital demands and collective capabilities in low-income households. Information, Commun Soc. 2025;28:1–19. doi: [10.1080/1369118X.2024.2449007](https://doi.org/10.1080/1369118X.2024.2449007)
22. Pope C, Maclellan J, Prichard J, Turnbull J. The remarkable invisibility of NHS 111 online. Sociol Health Illn. 2022;45. doi: [0.1111/1467-9566.13591](https://doi.org/10.1111/1467-9566.13591)
23. Ragnedda M, Ruiu ML. Digital Capital: A Bourdieusian Perspective on the Digital Divide. Emerald Group Publishing; 2020.
24. Ragnedda M, Ruiu M, Addeo F. The self-reinforcing effect of digital and social exclusion: The inequality loop. Telemat Informatics. 2022;72:101852. doi: [10.1016/j.tele.2022.101852](https://doi.org/10.1016/j.tele.2022.101852)
25. Ruiz-Figueroa I, Minguela Recover MÁ, Gómez P. A Social Work Analysis of Facilitators of and Barriers to Adopting Technology in Older Adults: A Systematic Literature Review. J Gerontol Soc Work. 2024;67:1–21. doi: [10.1080/01634372.2024.2339977](https://doi.org/10.1080/01634372.2024.2339977)
26. Rybczynska-Bunt S, Byng R, Spitters S, Shaw SE, Jameson B, Greenhalgh T. The reflexive imperative in the digital age: Using Archer’s “fractured reflexivity” to theorise widening inequities in UK general practice. Sociol Heal Illn.2024;46:1772–91. doi: [10.1111/1467-9566.13811](https://doi.org/10.1111/1467-9566.13811)
27. Salemink K, Townsend L, Chapman P. The remapping of rural digitalisation: A just-rural narrative review. J Rural Stud. 2025;113. doi: [10.1016/j.jrurstud.2024.103499](https://doi.org/10.1016/j.jrurstud.2024.103499)
28. Sharma A, Ojha J. Analyzing the gender digital divide through powercube framework: A systematic literature review. Inf Dev. 2025; 41(2):145-162. doi: [10.1177/026666692513370](https://doi.org/10.1177/02666669251337064)
29. Sin J, Franz R, Munteanu C, Neves B. Digital Design Marginalization: New Perspectives on Designing Inclusive Interfaces. In: Proceedings of the 2021 CHI Conference on Human Factors Computing systems. 2021 May 6. P. 1-11. [doi: 10.1145/3411764.3445180](https://doi.org/10.1145/3411764.3445180)
30. Tsatsou P. Vulnerable people’s digital inclusion: intersectionality patterns and associated lessons. Information, Commun Soc. 2022;25:1475–94. doi: 10.1080/1369118X.2021.1873402
31. Western MJ, Smit ES, Gültzow T, Neter E, Sniehotta FF, Malkowski OS, et al. Bridging the digital health divide: a narrative review of the causes, implications, and solutions for digital health inequalities. Heal Psychol Behav Med. 2025;13:2493139. doi: 10.1080/21642850.2025.2493139
